# The incidence of myocardial injury following post-operative Goal Directed Therapy

**DOI:** 10.1186/1471-2261-7-10

**Published:** 2007-03-19

**Authors:** Rupert M Pearse, Deborah Dawson, Jayne Fawcett, Andrew Rhodes, R Michael Grounds, David Bennett

**Affiliations:** 1 Barts and The London School of Medicine and Dentistry, Queen Mary's University of London, UK; 2 Intensive Care Unit, St. George's Hospital, London, UK; 3 Anaesthetic Laboratory, 5th floor, 38 Little Britain, St. Bartholomew's Hospital, London. EC1A 7BE, UK

## Abstract

**Background:**

Studies suggest that Goal Directed Therapy (GDT) results in improved outcome following major surgery. However, there is concern that pre-emptive use of inotropic therapy may lead to an increased incidence of myocardial ischaemia and infarction.

**Methods:**

Post hoc analysis of data collected prospectively during a randomised controlled trial of the effects of post-operative GDT in high-risk general surgical patients. Serum troponin T concentrations were measured at baseline and on day 1 and day 2 following surgery. Continuous ECG monitoring was performed during the eight hour intervention period. Patients were followed up for predefined cardiac complications. A univariate analysis was performed to identify any associations between potential risk factors for myocardial injury and elevated troponin T concentrations.

**Results:**

GDT was associated with fewer complications, and a reduced duration of hospital stay. Troponin T concentrations above 0.01 μg l^-1 ^were identified in eight patients in the GDT group and six in the control group. Values increased above 0.05 μg l^-1 ^in four patients in the GDT group and two patients in the control group. There were no overall differences in the incidence of elevated troponin T concentrations. The incidence of cardiovascular complications was also similar. None of the patients, in whom troponin T concentrations were elevated, developed ECG changes indicating myocardial ischaemia during the intervention period. The only factor to be associated with elevated troponin T concentrations following surgery was end-stage renal failure.

**Conclusion:**

The use of post-operative GDT does not result in an increased incidence of myocardial injury.

## Background

The term Goal Directed Therapy (GDT) describes the use of intra-venous fluid and inotropic agents to increase cardiac output and related haemodynamic parameters to pre-determined levels. Several studies suggest that the use of GDT results in improved outcome, when commenced either before or after major surgery [1-6]. However, there has been some concern that the pre-emptive use of inotropic therapy in the absence of traditional indications for such treatment, may lead to an increased incidence of myocardial ischaemia and infarction. The incidence of ischaemic heart disease in patients undergoing major surgery is high [7], and it is possible that the use of pharmacological agents which increase myocardial work at a time of increased physiological stress may result in an imbalance of myocardial oxygen supply and demand. Patients who do not readily achieve the pre-determined haemodynamic goals may be at particular risk of such complications [8]. The absence of data describing the myocardial effects of GDT is an important obstacle to the wider use of this potentially beneficial treatment.

The use of cardiac troponin assays may provide a reliable marker of post-operative myocardial injury. The high sensitivity and specificity of these biochemical tests has allowed the identification of a group of patients who have sustained myocardial injury without detectable electrocardiograph (ECG) changes [9]. The use of such assays has greatly influenced the approach to the management of acute myocardial ischaemia [10]. Elevated serum troponin concentrations in the absence of ECG changes are commonly identified following major general surgery,[9] and are associated with poor outcome [11]. There is also a high incidence of elevated troponin concentrations in the critically ill [12,13], although it is unclear whether this is due to myocardial ischaemia or sepsis-related myocardial injury.

There is a need for more detailed information regarding the myocardial effects of GDT and the troponin assay would appear to be an ideal tool with which to evaluate these in more detail. The aim of this study was to investigate the effects of post-operative GDT on the incidence of myocardial injury, identified through the measurement of serum troponin T as well as conventional clinical means.

## Methods

This was a post hoc analysis of data collected prospectively during a randomised trial of the effects of GDT in patients undergoing major surgery. This study was approved by the Local Research Ethics Committee of St George's Healthcare National Health Service Trust. A detailed description of patient recruitment, clinical management and outcome data is provided in the original manuscript [4]. Exclusion criteria included acute myocardial ischaemia. Patients were monitored continuously during the eight hour intervention period. The GDT protocol required reductions in the dose of dopexamine in the event of tachycardia or myocardial ischaemia. Blood samples were taken at baseline and on the morning of the first and second post-operative days. Serum troponin T was measured by an automated technique in the chemical pathology laboratory at St George's Hospital (Elecsys 2010, Roche Diagnostics Ltd., Lewes, UK). Troponin T concentrations below 0.01 μg l^-1 ^were given a value of zero for the purposes of analysis. Patients were followed up for 60 days to identify complications which were defined prospectively.

Data are presented as mean (SD) where normally distributed and median (IQR) where not normally distributed. Normality was tested with the Kolmogorov-Smirnov test. Continuous data were analysed with the *t *test where normally distributed and the Mann Whitney U test where not normally distributed. Differences in physiological trends were assessed using repeated measures analysis of variance (ANOVA) with Bonferroni's correction. Univariate analysis was performed to test associations between potential risk factors for myocardial injury and elevated troponin T concentrations. Analysis was performed using GraphPad Prism version 4.0 for Windows (GraphPad Software, San Diego, USA) and significance was set at p < 0.05.

## Results

122 patients were recruited between November 2002 and August 2004. 62 patients were randomised to GDT and 60 patients to control treatment. Baseline data and management protocols are presented in detail in the original manuscript [4]. There were no significant differences between the groups with respect to baseline demographic data, type of surgery or inclusion criteria. Data describing the incidence of co-existing disease and pre-operative cardiac medication are presented in table 1. Surgical and anaesthetic management were also similar in the two groups. Fewer patients developed complications in the GDT group (27 patients [44%] vs. 41 patients [68%] RR 0.64 [0.46–0.89]; p = 0.007). The total number of complications was also lower in the GDT group. Median duration of hospital stay in the GDT group was significantly reduced (14 days [11–27] vs. 11 days [7–15]; p = 0.001). There was no difference in 28 day mortality (6 patients [9.7%] vs. 7 patients [11.7%]; p = 0.78).

**Figure 1:**
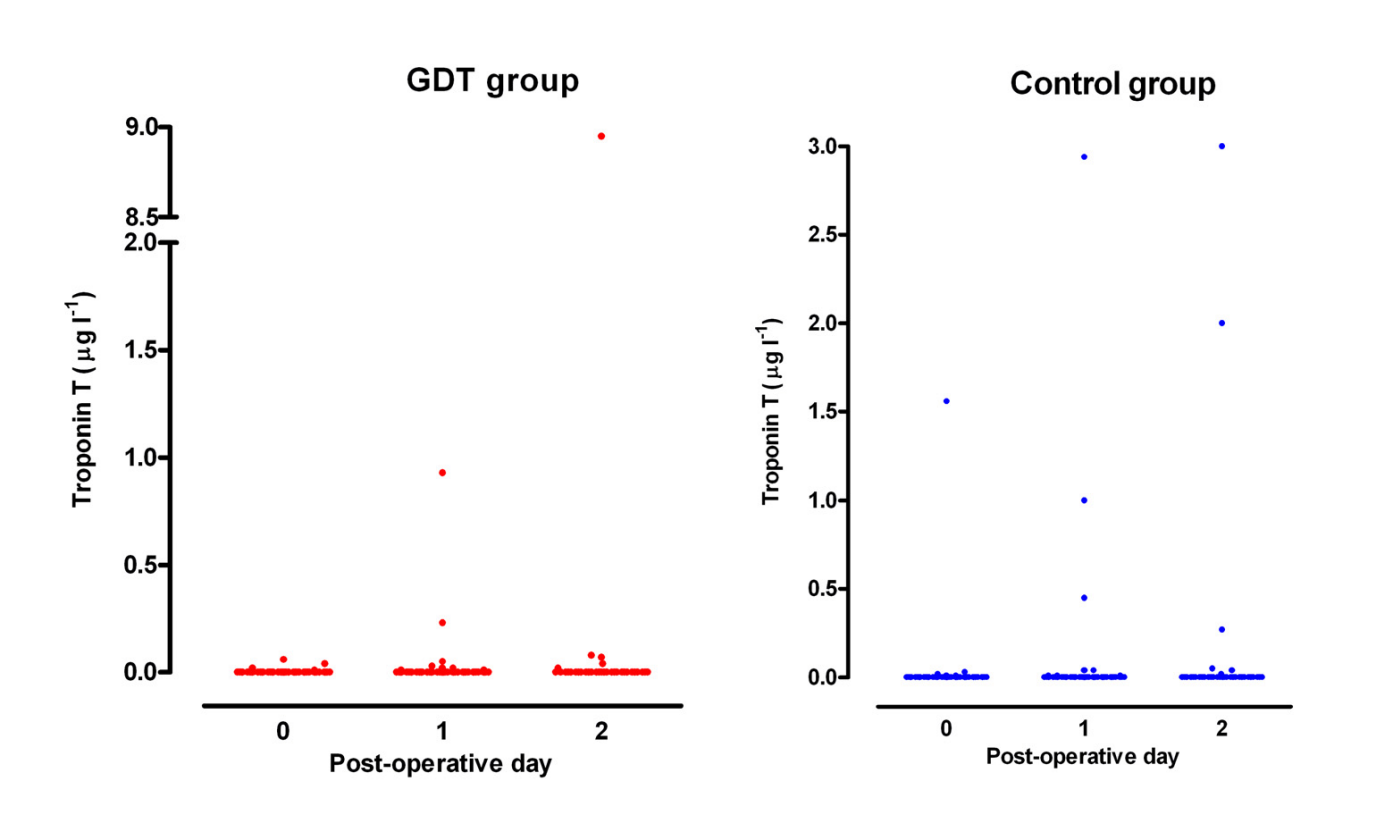
Post-operative troponin T measurements in patients randomised to the Control and Goal Directed Therapy (GDT) groups.

Serum troponin T concentrations greater than 0.01 μg l^-1 ^were identified in six patients in the control group and eight patients in the GDT group (figure 1). Troponin T concentrations above 0.05 μg l^-1 ^were identified in two patients in the control group and four patients in the GDT group. Troponin T was normal at baseline but subsequently increased to greater than 0.05 μg l^-1 ^in one patient in the control group and three patients in the GDT group. There were no overall differences in troponin T concentrations between the groups. The incidence of cardiovascular complications was also similar (table 2). Of the six control group patients, in whom there were elevated troponin concentrations, four had a pre-operative diagnosis of ischaemic heart disease and two were diagnosed with post-operative myocardial infarction. Of the eight GDT group patients, in whom there were elevated troponin concentrations, four had a pre-operative diagnosis of ischaemic heart disease. One patient developed septic shock, one suffered a brainstem stroke, one developed atrial fibrillation, one developed left ventricular failure and one developed cardiogenic shock with pulmonary oedema. None of the patients in whom troponin T concentrations were elevated, developed ECG changes indicating myocardial ischaemia during the eight hour intervention period. Univariate analysis was performed to establish which potential cardiac risk factors were associated with post-operative myocardial injury (defined by a troponin T concentration of 0.01 or above). The only characteristic associated with an elevated troponin concentration was end-stage renal failure (table 3). When those patients with elevated troponin T at baseline were excluded, there were no associations with any of the potential risk factors tested.

**Figure 2:**
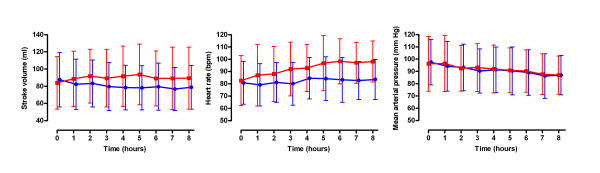
Haemodynamic data for goal directed therapy and control groups during the intervention period. Significant differences between groups: heart rate, stroke volume (both p < 0.0001, repeated measures ANOVA) but not mean arterial pressure (p = 0.87). Red squares: GDT group, Blue circles: control group. Data presented as mean (SD).

During the intervention period, the oxygen delivery index (DO_2_I) target of 600 ml min^-1 ^m^-2 ^was achieved by 49 patients in the GDT group (79%). This value was also attained spontaneously by 27 patients in the control group (45%) (p = 0.0002). The difference in DO_2_I between the groups resulted from a comparable difference in cardiac index, which resulted from greater heart rate (p = 0.0001) and stroke volume (p = 0.0001) in the GDT group, whilst mean arterial pressure remained similar in the two groups throughout the intervention period (figure 2). Post-operative haemodynamic interventions are summarised in table 4. One patient in the control group received dopexamine at a dose of 1 μg kg^-1 ^min^-1 ^on the instruction of clinical staff. Despite receiving maximal therapy allowed by the protocol, 13 patients in the GDT group did not achieve the goal for DO_2_I. In seven of these patients the dose of dopexamine was reduced, in six patients this was because of tachycardia and in one because of myocardial ischaemia. A similar number of patients in each group received epinephrine in order to maintain cardiac index above 2.5 l min^-1 ^m^-2 ^as dictated in the study protocol.

## Discussion

This analysis suggests that the use of post-operative GDT does not result in an increased incidence of myocardial injury. The incidence of cardiovascular complications identified by traditional clinical criteria was also similar in the two groups. However, the heart rate in the GDT group was significantly higher than that of the control group. The study protocol required a reduction in the dose of dopexamine in the event of tachycardia or myocardial ischaemia. This aspect of study design may explain the absence of cardiac sequelae in patients in the GDT group. Patients in the GDT group received an average of 700 ml more intra-venous fluid and the majority also received low dose dopexamine. It is possible that the use of alternative treatment goals or interventions may have been associated with different findings. The only risk factor identified by univariate analysis as being associated with elevated serum troponin T concentration was end-stage renal failure. Multivariate analysis was therefore not performed. Whilst elevated cardiac troponin concentrations are a recognised feature of end-stage renal failure [14], this finding still has significant prognostic value [15].

Despite long standing concerns that GDT may result in myocardial ischaemia, few studies have evaluated this hypothesis in detail. One study of 89 surgical patients, did not identify an increased incidence of myocardial infarction associated with the use of catecholamines to achieve haemodynamic goals [16]. A small study in vascular surgical patients suggested the use of dopexamine to increase oxygen delivery may not result in increased myocardial oxygen demand [17]. Whilst a study of the use of GDT in a mixed group of critically ill patients suggested that continued attempts to achieve haemodynamic goals in unresponsive patients may increase the incidence of myocardial ischaemia [8].

Although the current data suggests that GDT does not result in an increased incidence of myocardial injury, there are a number of weaknesses in the analysis presented. This was a post hoc analysis of data collected prospectively during a small, single centre trial. The study was not specifically designed to test the association between potential cardiac risk factors and post-operative myocardial injury. The low incidence of post-operative myocardial injury identified in this study may explain the fact that only one potential risk factor for myocardial injury was associated with an elevation in troponin T concentration. Acute myocardial ischaemia was a specific exclusion criterion, but no attempts were made to standardise cardiac medications prior to surgery or stratify patients for the presence of myocardial disease. There were no overall differences in pre-operative cardiac medication or co-morbidity. However, five patients in the control group were taking digoxin prior to surgery compared to none in the GDT group. The indications for these prescriptions are not known. The use of cardiac troponins appears to be a very reliable method of identifying post-operative myocardial injury [9,11]. However, increased plasma concentrations of troponins may relate not to acute myocardial ischaemia but to other mechanisms of injury such as sepsis [13]. It appears that this may also have been the case in the present study. Post-operative elevations in troponin concentrations in the absence of ECG changes have been identified previously in general surgical patients [9], and have prognostic significance [11]. Troponin assays would therefore appear to be the most effective method of identifying myocardial injury resulting from the use of GDT.

## Conclusion

The use of post-operative GDT does not appear to result in an increased incidence of myocardial injury detected through clinical, electrocardiographic or biochemical means. GDT was not associated with an increased incidence of clinically significant cardiovascular complications.

## Key messages

• Goal directed therapy (GDT) appears to improve outcome following major surgery

• There is concern that the pre-emptive use of inotropic therapy may result in an increased incidence of myocardial ischaemia

• This analysis suggests GDT does not affect the incidence of myocardial injury detected either through biochemical or clinical methods

## Abbreviations

DO_2_I oxygen delivery index

ECG electrocardiograph

GDT Goal Directed Therapy

IQR inter-quartile range

SD standard deviation

## Competing interests

The author(s) declare that they have no competing interests.

## Authors' contributions

RP, DD and JF were responsible for data collection. RP performed the post hoc analysis. All authors read and approved the final manuscript.

## Pre-publication history

The pre-publication history for this paper can be accessed here:



## Figures and Tables

**Table 1:** Incidence of common co-existing diseases and pre-operative cardiac medication in the goal directed therapy and control groups.

**Disease**	**Control**	**GDT**
Hypertension	35 (58%)	34 (55%)
Ischaemic heart disease	23 (38%)	18 (29%)
Chronic obstructive pulmonary disease	8 (13%)	8 (13%)
Diabetes	8 (13%)	6 (10%)
End-stage Renal Failure	6 (10%)	7 (11%)
Obesity	2 (3%)	3 (5%)
Stroke	7 (12%)	2 (3%)
**Drug**		
β-blockade	15 (25%)	14 (23%)
ACE inhibitor	18 (30%)	18 (29%)
Calcium channel antagonist	14 (23%)	16 (26%)
Diuretics	19 (32%)	14 (23%)
Digoxin	5 (8%)	0 (0%)

Data presented as absolute value (%)

**Table 2:** Post-operative cardiovascular complications in Goal Directed Therapy (GDT) and control groups.

**Complication**	**Control group**	**GDT group**
Arrythmia	9	5
Acute heart failure	4	4
Myocardial infarction	3	0
Stroke	1	2
Severe sepsis/septic shock	9	1

Data presented as absolute values.

**Table 3:** Univariate analysis of potential risk factors for post-operative myocardial injury and elevated troponin T concentration.

**Potential risk factor for post-operative myocardial injury**	**Troponin T >0.01 at any time**		**Troponin T >0.01 when normal at baseline**	
	**n**	**p**	**n**	**p**

Goal directed therapy	10 (16%)	>0.99	6 (10%)	>0.99
Ischaemic heart disease	7 (18%)	0.80	3 (8%)	>0.99
End-stage renal failure	6 (46%)	0.008*	1 (8%)	>0.99
Hypertension	13 (19%)	0.30	7 (10%)	0.75
Diabetes	3 (23%)	0.45	1 (8%)	>0.99
COPD	2 (13%)	>0.99	2 (13%)	0.64
Obesity	1 (20%)	>0.99	0 (0%)	-
Stroke	0 (0%)	-	0 (0%)	-
Vascular surgery	5 (18%)	0.78	3 (11%)	0.71
Pre-operative cardiac medication	12 (17%)	0.80	6 (9%)	>0.99

* signifies statistical significance; COPD, chronic obstructive pulmonary disease

**Table 4:** Haemodynamic management of patients in Goal Directed Therapy (GDT) and control groups during the intervention period.

	**Control group**	**GDT group**	**p**
**Intra-venous fluid**			
Crystalloid (ml)	960 (335)	930 (221)	0.39
Colloid (ml)	1204 (898)	1907 (878)	<0.0001
Blood (ml)	0 (0 – 485)	125 (0 – 734)	0.09
**Vasoactive drugs**			
No. of patients receiving dopexamine	1 (2%)	55 (89%)	<0.0001
Dose of dopexamine (μg kg^-1 ^min^-1^)	0 (0 – 0)	0.75 (0.5 – 1.0)	<0.0001
No. of patients receiving epinephrine	3 (5%)	2 (3%)	0.68

Data presented as mean (SD), median (IQR) or absolute value (%).
